# Illuminating the impact of CD38-induced adenosine formation in B-cell lymphoma

**DOI:** 10.1038/s41598-024-82800-1

**Published:** 2025-01-13

**Authors:** Shams ElDoha Galal ElDin Zaiema, Heba Mohamed Saber Hafez, Diaa El-Din Moussa Sherif Abou El-Ela, Rawda Ahmed Alaaeldin Ahmed Mohamed Saad

**Affiliations:** 1 Department of Clinical and Chemical Pathology, Ain shams University, Cairo, Egypt; 2 Department of Haematology – Internal Medicine, Ain shams University, Cairo, Egypt; 3 Department of Clinical Oncology and Nuclear Medicine, Ain shams University, Cairo, Egypt

**Keywords:** CD38 expression, Extracellular ADO, Mature B-cell lymphoma, Chronic lymphocytic leukemia, B-Non-Hodgkin lymphoma, PD-1/PD-L1 axis, A promising therapeutic immune target, Oncology, Cancer, Haematological cancer

## Abstract

The expression of CD38 by cancer cells may mediate an immune-suppressive effect by producing Extracellular Adenosine (ADO) acting through G-protein-coupled cell surface receptors on cellular components and tumor cells. This can increase PD-1 expression and interaction with PD-L1, suppressing CD8 + cytotoxic T cells. This study examines the impact of heightened CD38 expression and extracellular ADO on various hematological and clinical parameters in patients with mature B-cell lymphoma, alongside their correlation with the soluble counterparts of the PD-1/PD-L1 axis. Our study was conducted on 90 patients, CD38-positive and CD38-negative (measured by flow cytometry), with mature B-cell lymphoma divided into CLL and B-NHL subtypes. Their serum ADO, soluble PD-1, and PD-L1 levels were measured using a sandwich ELISA. Our study revealed a positive correlation between CD38 expression, sADO, sPD-1, and sPD-L1 in mature B-cell lymphoma patients. CD38-positive patients had higher sADO, sPD-1, and sPD-L1 levels. Higher CD38 expression and extracellular ADO negatively affected HB level and PLT count and positively correlated with the higher risk stratification in mature B-cell lymphoma patients. This study explored the potential impact of CD38 expression and elevated extracellular ADO on B-cell lymphoma alongside their link with the PD-1/PD-L1 axis. Our findings underscore the influence of extracellular ADO on the neoplastic process of mature B-cell lymphoma. We also propose targeting the CD38-induced-ADO formation pathway, which could serve as a promising therapeutic immune target with multifaceted effects within mature B-cell neoplasms.

## Introduction

Mature B-cell lymphomas are a diverse group of cancers that originate from B cells. These include Chronic Lymphocytic Leukemia (CLL) and non-Hodgkin’s Lymphomas (NHL). The diseases are characterized by various immune system dysfunctions^1^.

Research studies have underscored the crucial role of the PD-1/PD-L1 axis dysregulation, an essential immune checkpoint in mature B-cell lymphomas, in immune escape and tumor progression^2^. Malignant tumor B-cells have been found to upregulate PD-L1 expression, enabling immune evasion by engaging PD-1 on T cells. This leads to T cell exhaustion and impaired antitumor immune responses. The urgency to find a solution to this immune escape mechanism is evident^3^. Because of these properties, PD-1/PD-L1 axis blockage can potentially be a promising target for immunotherapy for mature B-cell lymphomas^4,5^.

Recent preclinical trials have revealed a significant breakthrough resistance to anti-PD-1/PD-L1 immunotherapy in various solid tumors, including hepatocellular carcinoma (HCC) and multiple myeloma (MM), is instigated by the up-regulation of CD38 on tumor cells^6^.

CD38 is a multifunctional molecule that functions as a transmembrane signaling receptor and an ectoenzyme. It plays essential roles in cell adhesion, calcium regulation, and signal transduction^7^. This transmembrane glycoprotein, expressed on the surface of a substantial percentage of mature B-cell lymphomas (B-NHL and B-CLL), is associated with poor prognosis and clinical outcomes. The potential of CD38 as a therapeutic target is a compelling avenue for further exploration^8,9^.

Researchers have postulated that the resistance to anti-PD-1/PD-L1 immunotherapy in cancer cells is orchestrated by CD38-mediated-ADO production, a process known as CD38-ADO-mediated immune suppression^10^. Their findings suggest that Extracellular ADO amplifies PD-1 expression in CD8 + T cells, triggering all-trans retinoid acid and IFN-β that negatively regulate intra-tumor CD8 + cytotoxic T cells, leading to their exhaustion and subsequent resistance to anti-PD-1 blockage^11^.

Adenosine (ADO), a potent signaling molecule, activates cellular signaling pathways through a family of four G protein-coupled adenosine receptors (AR): A1, A2A, A2B, and A3^12^. Under normal conditions, extracellular ADO levels remain low due to the balanced processes of production from ATP/AMP by ectonucleotidases (CD39, CD73), rapid cellular uptake by nucleoside transporters, and degradation by adenosine deaminase (ADA)^13^. However, extracellular ADO levels surge in the tumor microenvironment due to the release of intracellular ADO from damaged cells or in response to hypoxia, inflammation, and tissue injury. This surge significantly disrupts tissue homeostasis and hematopoiesis by influencing cellular activation, differentiation, and cell cycling^13–15^.

Nevertheless, little is known about the exact effect of enhanced CD38 expression and extracellular ADO production or their mediated immune-suppressive impact linked to the PD-1/PD-L1 axis in mature B-cell lymphomas. Analyzing these immune molecules is needed to elucidate the underlying mechanisms of resistance and identify predictive biomarkers to guide patient selection and optimize treatment strategies.

In this study, we aim to analyze the effects of elevated CD38 expression and extracellular ADO on different hematological and clinical parameters in mature B-cell lymphoma patients and their link to the soluble counterparts of the PD-1/PD-L1 axis.

## Patients and methods

### Patients

This is a cross-sectional study done on mature B-cell lymphoma patients. All patients were recruited from Ain Shams University Hospitals, Oncology Hospital, and the Clinical Hematology departments of Internal Medicine Hospital, Cairo, Egypt. All patients’ clinical history data were collected from their hospital files.

#### Inclusion criteria

Eligible 90 newly diagnosed mature B-cell lymphoma patients were recruited prospectively into the study; patients’ ages ranged from 18 to 80 years. Based on *the 4th and 5th editions of the World Health Organization classification of haematolymphoid tumors: lymphoid neoplasms*, using the integration of bone marrow and peripheral blood morphology, IPT, and cytogenetics studies, they were subcategorized into the CLL patients’ group (*n* = 48) and the B-NHL patients’ group (*n* = 42). The final diagnosis of the 42 B-NHL patients: Diffuse large B-cell lymphoma (DLBCL) was found in 14 patients (33.3%), Follicular lymphoma (FL) in 8 patients (19%), Mantle lymphoma (ML) in 6 patients (14.3%), Marginal zone lymphoma (MZL) in 5 patients (11.9%), Burkitt’s lymphoma in 2 patients (4.7%), Lymphoplasmacytic lymphoma in 1 patient (2.3%), and 6 patients (14.2%) were not classifiable.

*The Binet staging system* for CLL was used for staging and risk stratification in CLL patients. Patients were categorized into Stage A (Low risk), B (Intermediate risk), and Stage C (High risk).

*Our study used the Ann Arbor staging system* to stage B-NHL patients. All were chosen with BM involvement (Stage IV) to make the comparison more credible regarding the degree of CD38 expression % by flow cytometry.

*The International Prognostic Index and ECOG performance scale* (based on a patient’s age, serum lactate dehydrogenase (LDH) concentration, Eastern Cooperative Oncology Group (ECOG) performance status, Ann Arbor clinical stage, and the number of involved extranodal disease sites) were used to stratify B-NHL patients into Low-risk, intermediate-risk (intermediate-low and intermediate-high), and high-risk patients.

#### Exclusion criteria

This research excluded patients with solid tumors and other hematological malignancies or exposed to leukemogenic therapies.

### Methods

#### Sampling method

- Each subject provided a 10 cm non-fasting peripheral blood venous sample to obtain a set of three blood tubes: one tube EDTA and two anticoagulant-free tubes.
- A bone marrow sample of 3 ml was obtained from each patient.

#### All participants included in this study were subjected to the following procedures

- Full history taking and clinical examination, including age, sex, and presence of lymphoma-associated symptoms and signs, B symptoms, hepatomegaly, splenomegaly, and lymphadenopathy.
- Routine diagnostic workups for mature B-cell lymphomas, including:
  Complete blood count (CBC) using an EDTA blood sample analyzed on a Sysmex XN-1000 autoanalyzer (Sysmex Corporation, Kobe, Japan). Examination of Leishman-stained peripheral blood smears, focusing on lymphocyte count and morphology, Hb concentration, and platelet count.
  Evaluation of BM infiltration by examination of Leishman-stained BM smears to evaluate the percentage and morphology of lymphocytes (small, intermediate, or large sized or with blastoid morphology ± nuclear and cytoplasmic vacuolation).
  Immunophenotyping analysis for mature B-cell lymphomas panel using an EDTA sample of bone marrow or peripheral blood using a Beckman Coulter NAVIOS flow cytometer (Coulter Electronics, Hialeah, FL, USA). The populations analyzed were selected using primarily the side scatter against the CD45 gate, then the CD45 against the CD19 gate. Our populations showed CD19 and CD45 bright or intermediate positivity (mature B-lymphocytes). Secondly, the following markers were assessed: CD20, CD79b, FMC7, CD5, CD23, CD10, surface Ig ab, Kappa or Lambda light chain restriction, CD22, CD49, CD38, CD56, CD11C, CD200, CD25, CD103 and HLA-DR. The cutoff for these markers positivity were > 20% of the cells expressing it.
  FISH analysis was performed on Heparin bone marrow or peripheral blood samples for CCND1, c-MYC, BCL2, BCL6, 14q32 rearrangement, and P53 (17p13) deletion.
  Biochemical profile: LDH, Liver and kidney function tests, uric acid…etc.
- Radiological investigation: including CT of neck, chest, abdomen, and pelvis and PET/CT.
- Lymph node biopsy and histopathology if needed.
- Assay of serum soluble PD-1 (sPD-1), PD-L1 (sPD-L1), and ADO (sADO) were assayed by a commercially available sandwich ELISA kit using an anticoagulant-free tube separated by centrifugation at 1000×g for 15 min. The assay kit for Human PD-1 (Bioassay Technology Laboratory, E4711Hu, China), Human PD-L1 (Bioassay Technology Laboratory, E2153Hu, China), and Human ADO (Bioassay Technology Laboratory, EA0187Hu, China), assays were done according to manufacturer’s instruction. Due to the minimal half-life of ADO in vitro and in vivo, the samples were collected under aseptic conditions and immediately sent to the laboratory. Serum samples, after prompt separation from cells or clots, were kept tightly stoppered and were stored at -20 °C within less than one hour of collection till examination.

#### Administrative and ethical design

Based on the ethical committee regulations of Ain Shams University, each participant supplied written informed consent to participate in the study before being enrolled. Confidentiality and privacy were maintained throughout the entire study.

#### Statistical methods

The Statistical Package for Social Science (IBM SPSS version 23) was used in this research to analyze collected data. The quantitative data was shown as Mean ± SD and ranges when they displayed a parametric distribution as Median with IQR when they displayed a non-parametric distribution. For qualitative data, they were shown as % and numbers. The groups were compared using the Chi-square and Fisher exact tests. The independent t-test used quantitative data and parametric distribution to compare two groups, whereas the Mann-Whitney test was used for non-parametric distribution. The allowable margin of error was set at 5%, while the confidence interval was set at 95%. As a result, the P-value was considered significant at 0.05.

## Results

### Demographic data and characteristics of all the studied mature B-cell lymphoma patients (including CLL and B-NHL)

Data are presented in Table 1.

**Table 1:** Demographic data and characteristics of all the studied mature B-cell lymphoma patients.

Demographic data of mature B-cell lymphoma		No. = 90
Age (Years)	Mean ± SD	55.57 ± 14.95
	Range	23–80
Sex	Females	25 (27.8%)
	Males	65 (72.2%)
Category/Diagnosis	CLL	48 (53.3%)
	B-NHL	42 (46.7%)
WBC (x10^3^/ul)	Median (IQR)	55 (20–94)
	Range	3.01 - 531
LYMPH (x10^3^/ul)	Median (IQR)	43.5 (16–82)
	Range	2 – 480
HB (g/dL)	Mean ± SD	9.71 ± 2.62
	Range	4.2 – 16.1
PLT (x10^3^/ul)	Median (IQR)	120 (60–171)
	Range	10 – 576
BM.LYM (%)	Mean ± SD	74.46 ± 19.38
	Range	10 – 98
sPD-L1(ng/L)	Median (IQR) Range	155 (104.5- 391.20) 21.51–1600
sPD-1(ng/L)	Median (IQR) Range	149 (98.68–270) 19.38–1300
sADO (ng/ml)	Median (IQR) Range	694 (536–986) 73-1700

### Studying the impact of elevated CD38 expression % and extracellular ADO production in all mature B-cell lymphoma patients

#### Studying the effect of CD38 expression % on mature B-cell lymphoma patients by comparing CD38-negative Vs. CD38-positive patients regarding demographic data and characteristics

**Fig. 1:**
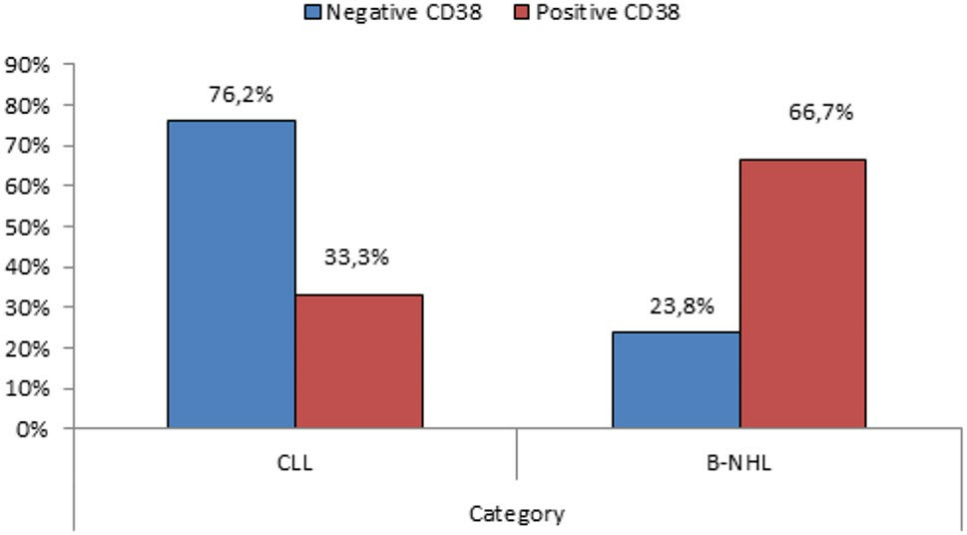
Comparison between CLL and B-NHL groups regarding CD38 expression %.

**Fig. 2:**
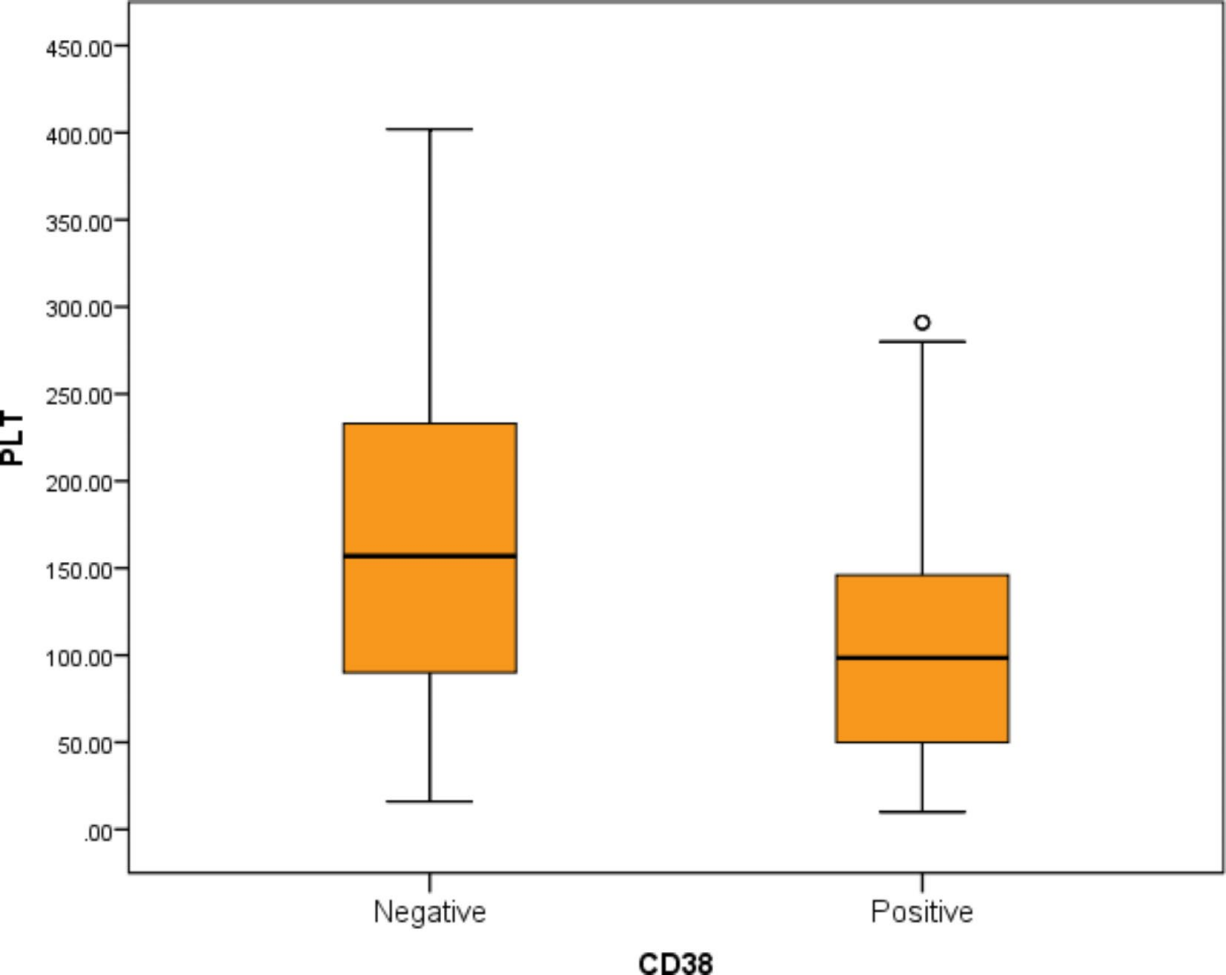
Comparison between CD38 positive and CD38 negative mature B-cell lymphoma patients regarding PLT count.

Using the cutoff ≥ 20% for CD38 expression by flow cytometry as an indicator of positivity, mature B-cell lymphoma patients were divided into CD38 positive (No. = 48) and CD38 negative (No. = 42) patients. Our study revealed a statistically significant difference regarding CD38 expression % in CLL and B-NHL groups (P-value < 0.01). In the CLL group, only 33% were positive for CD38, compared to 76.2% who were negative. On the contrary, in B-NHL, 66.7% were positive for CD38 compared to 23.8% who were negative (Fig. 1). Besides, sADO, sPD-L1, and sPD-1 serum levels were significantly higher in the CD38-positive patients when compared to the CD38-negative patients (P-value < 0.01). Finally, the platelets count showed a statistically significant decrease in the CD38-positive patients when compared to the CD38-negative patients (P-value < 0.01) (Fig. 2, Table 2).

**Table 2:** Studying the effect of CD38 expression % on mature B-cell lymphoma patients regarding demographic, hematological, and clinical characteristics:

Mature B-cell lymphoma		CD38 Negative	CD38 Positive	*P*-value
		No. = 42	No. = 48	
Age (years)	Mean ± SD	56.5 ± 8.9	54.7 ± 18.8	0.563•
	Range	40–69	23–80	
Sex	Females	12 (28.6%)	13 (27.1%)	0.875*
	Males	30 (71.4%)	35 (72.9%)	
Category/diagnosis	CLL	32 (76.2%)	16 (33.3%)	0.000*
	B-NHL	10 (23.8%)	32 (66.7%)	
WBC (x10^3^/ul)	Median (IQR)	59 (27.3–117)	50 (15.8–70)	0.138‡
	Range	9.3–531	3.01–428	
LYMPH (x10^3^/ul)	Median (IQR)	48 (20–97)	38.5 (14–61)	0.156‡
	Range	6–480	2–360	
HB (g/dl)	Mean ± SD	10.23 ± 2.91	9.26 ± 2.26	0.078•
	Range	4.2 – 16.1	4.6 – 14	
PLT (x10^3^/ul)	Median (IQR)	157 (90–233)	98.5 (50–146)	0.003‡
	Range	16–402	10–576	
BM.LYM (%)	Mean ± SD	76.14 ± 18.16	72.98 ± 20.46	0.443•
	Range	21 – 98	10 – 97	
P53 mutation	Negative Positive	8 (72.7%) 3 (27.3%)	7 (53.8%) 6 (46.2%)	0.341*
sPD-L1 (ng/L)	Median (IQR)	116.7 (68.25–240)	232.35 (153.75–452.75)	0.000‡
	Range	21.51–1427	100.9–1600	
sPD-1 (ng/L)	Median (IQR)	97.74 (47–135)	217.5 (156–661)	0.000‡
	Range	19.38–680	110–1300	
sADO (ng/ml)	Median (IQR)	530 (343–614)	835.5 (723–1332)	0.000‡
	Range	73–777	553–1700	
Risk stratification	Low	13 (31.0%)	9 (18.8%)	0.095*
	Intermediate	18 (42.9%)	16 (33.3%)	
	High	11 (26.2%)	23 (47.9%)	

*P* > 0.05: non-significant (NS); *P* < 0.05: significant (S); *P* < 0.01: highly significant (HS).

‡: Mann-Whitney test; •: Independent t-test; *: Chi-square test.

#### Studying the independent effect of lower vs. higher sADO level (regardless of CD38 expression %) on other studied parameters in mature B-cell lymphoma patients

**Fig. 3:**
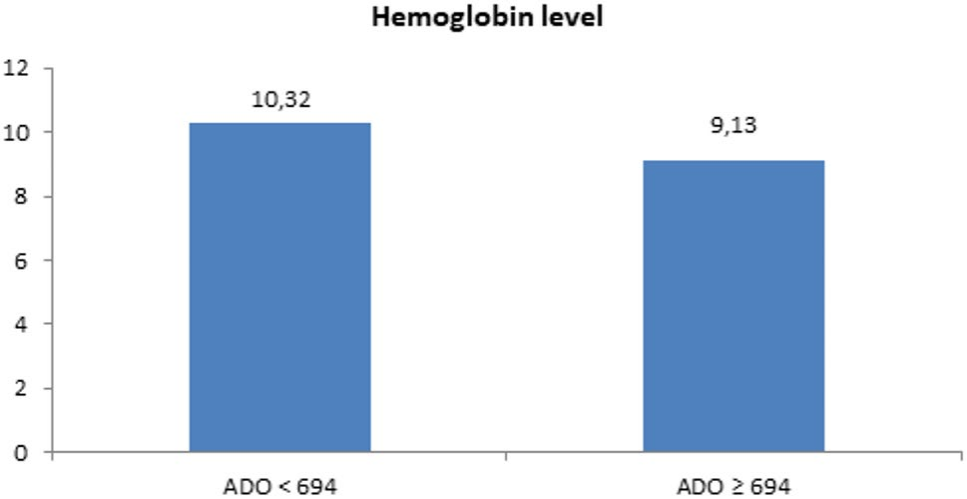
Comparison between lower vs. higher sADO level sub-groups of mature B-cell lymphoma patients regarding HB level.

**Fig. 4:**
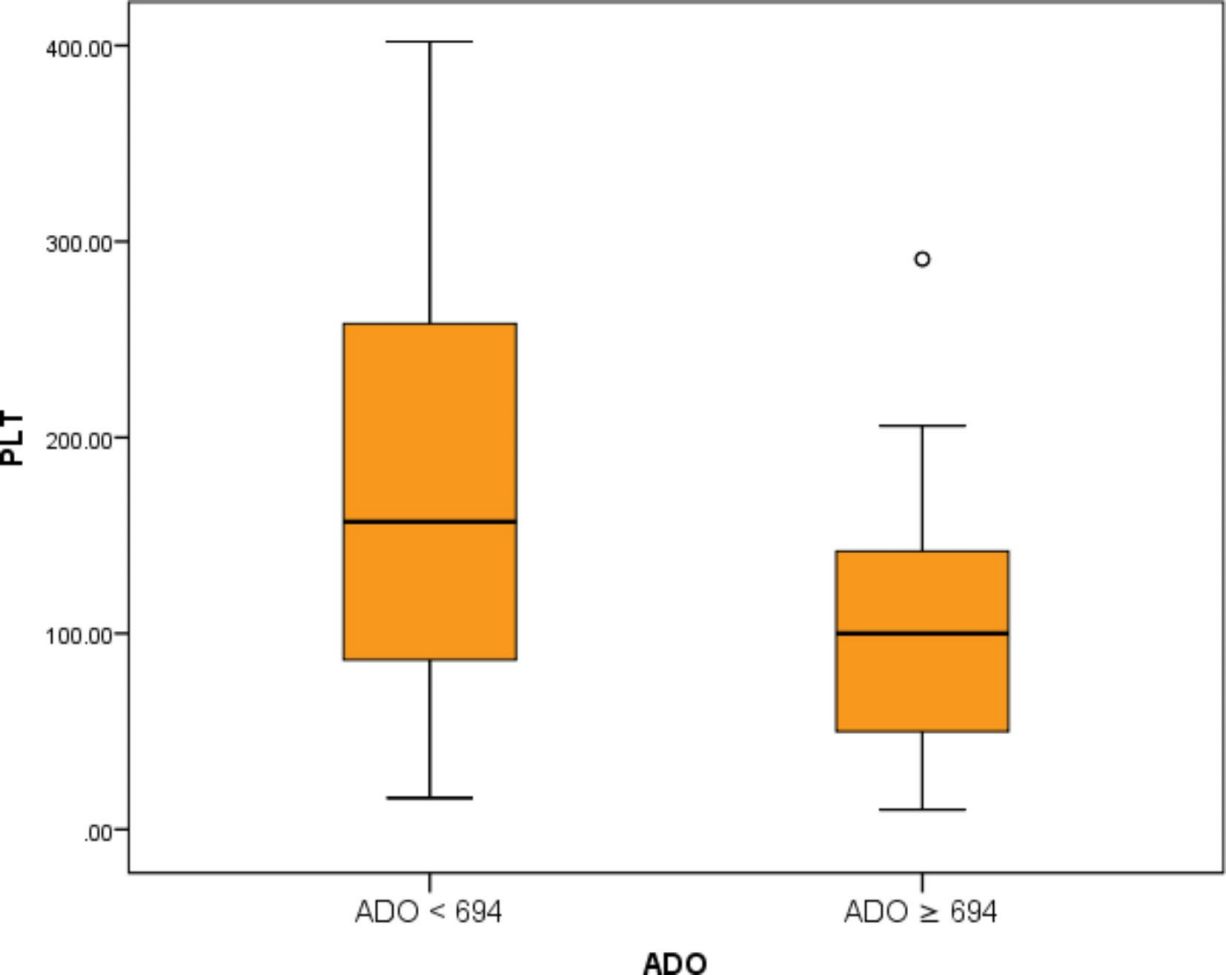
Comparison between lower vs. higher sADO level sub-groups of mature B-cell lymphoma patients regarding PLT count.

**Fig. 5:**
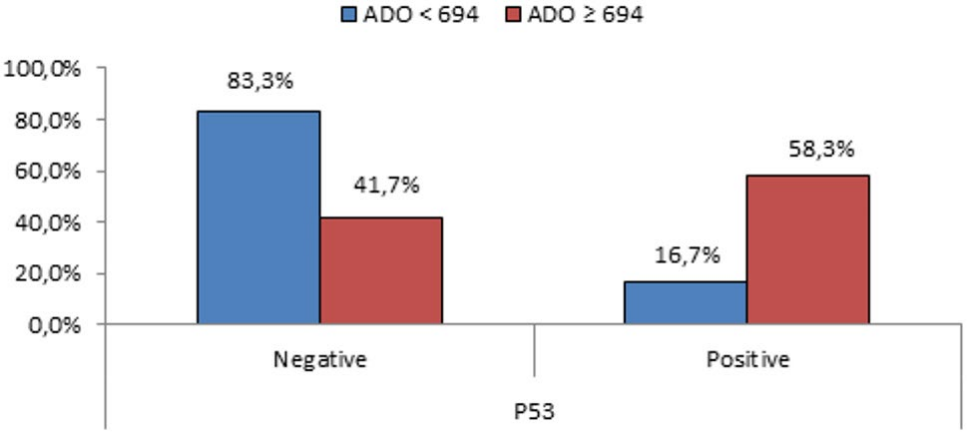
Comparison between lower vs. higher sADO level sub-groups of mature B-cell lymphoma patients regarding P53 deletion.

We used the mean of sADO level (694 ng/ml) in our study population to divide the mature B-cell lymphoma patients into two sub-groups: one with sADO < 694 ng/ml and another with sADO ≥ 694 ng/ml (Table 3). Comparison between these two groups regarding demographic data and characteristics showed that the sub-group with sADO ≥ 694 ng/ml had a statistically significant increase in CD38 expression % (P-value < 0.01), sPD-1 (P-value < 0.01), sPD-L1 (*P* < 0.05), and a statistically higher number of patients with P53 deletion (*P* < 0.05). Also, this group showed a statistically lower HB level (*P* < 0.05) and PLT count (P-value < 0.01) (Figs. 3, 4 and 5).

**Table 3:** Studying the independent effect of higher vs. lower sADO levels in mature B-cell lymphoma patients.

Mature B-cell lymphoma		sADO < 694 ng/ml	sADO ≥ 694 ng/ml	P-value
		No. = 44	No. = 46	
Age(years)	Mean ± SD	56.23 ± 10.13	54.93 ± 18.52	0.684•
	Range	23–69	23–80	
Sex	Females	13 (29.5%)	12 (26.1%)	0.714
	Males	31 (70.5%)	34 (73.9%)	
WBC(x 10^3^/ul)	Median (IQR)	58 (27.3–112.5)	50 (15.4–70)	0.248≠
	Range	3.5–831	3.01–589	
LYMPH(x 10^3^/ul)	Median (IQR)	48 (19.5–96.5)	32.5 (15–58)	0.218≠
	Range	2.4–165	2–538	
HB(g/dl)	Mean ± SD	10.32 ± 2.84	**9.13** ± 2.27	0.031•
	Range	4.2–16.1	4.2–14	
PLT(x 10^3^/ul)	Median (IQR)	157 (86.5–258)	**100** (50–142)	0.002≠
	Range	16–402	10–576	
BM.LYM(%)	Mean ± SD	74.61 ± 18.1	74.3 ± 20.73	0.940•
	Range	21–95	10–98	
P53 deletion	Negative	10 (83.3%)	5 (41.7%)	0.035*
	Positive	2 (16.7%)	**7** (58.3%)	
CD38 degree	Median (IQR)	4.5 (1–8)	58 (41–87)	0.000≠
	Range	0–99	0.1–100	
sPD-L1(ng/L)	Median (IQR)	126.2 (70.91–395.15)	173.98 (140.6–391.2)	0.017≠
	Range	21.51–1427	50–1600	
sPD-1(ng/L)	Median (IQR)	99.34 (54–153.5)	197 (148–312)	0.000≠
	Range	19.38–1070	37–1300	
Risk stratification	Low	15 (34.1%)	7 (15.2%)	0.088*
	Intermediate	16 (36.4%)	18 (39.1%)	
	High	13 (29.5%)	21 (45.7%)	

P-value > 0.05: non-significant; P-value < 0.05: significant; P-value < 0.01: highly significant. *: Chi-square test; •: Independent t-test; ≠: Mann-Whitney test.

#### Studying sADO, sPD-L1, and sPD-1 correlations with the other studied parameters among all mature B-cell lymphoma patients

**Fig. 6:**
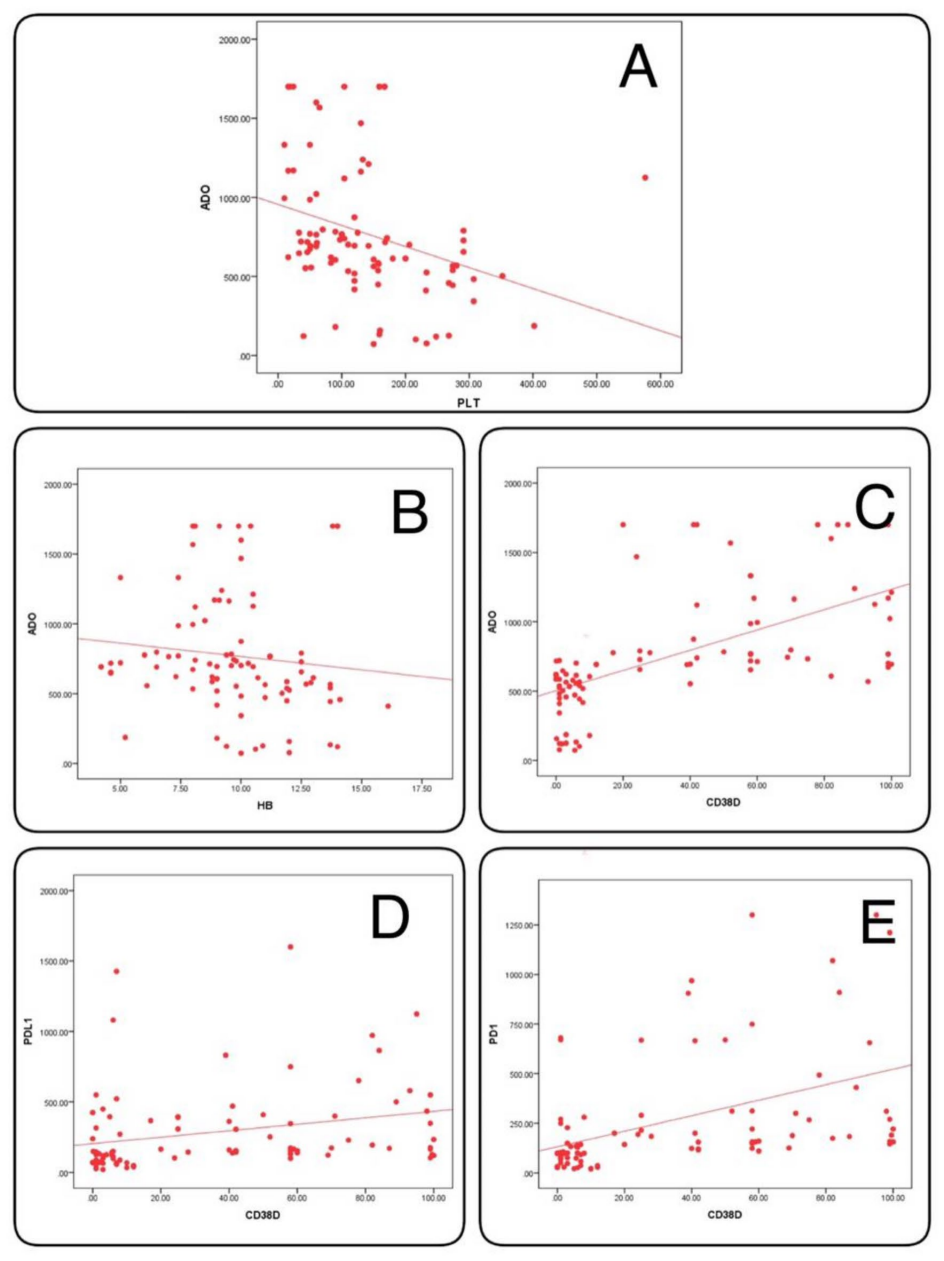
Showing correlations between sADO and PLT count (**A**), sADO and HB level (**B**), sADO and CD38 expression % (**C**), PD-L1 and CD38 expression % (**D**), and PD-1 and CD38 expression % (**E**) in mature B-cell lymphoma patients.

Our data revealed a statistically significant positive correlation between CD38 expression % and sPD-L1, sPD-1, and sADO in all mature B-cell lymphoma patients (P-value < 0.01). Meanwhile, sADO showed a significant negative correlation with HB level and Platelets count (P-value < 0.01) (Table 4, Fig. 6).

**Table 4:** Studying sADO, sPD-L1, and sPD-1 correlations with the other studied parameters among all mature B-cell lymphoma patients:

Mature B-cell lymphoma	sADO		sPD-1		sPD-L1	
	*r*	*p*-value	*r*	*p*-value	*r*	*p*-value
CD38 Degree	0.702**	**0.000**	0.565**	**0.000**	0.425**	**0.000**
sPD-L1 (ng/L)	0.318**	**0.002**	0.600**	**0.000**		
sPD-1(ng/L)	0.498**	**0.000**			0.600**	**0.000**
sADO (ng/ml)			0.498**	**0.000**	0.318**	**0.002**
Age (years)	0.042	0.691	0.022	0.840	0.136	0.201
WBC (x10^3^/ul)	− 0.185	0.080	− 0.010	0.928	− 0.102	0.339
LYMPH (x10^3^/ul)	− 0.180	0.090	− 0.050	0.639	− 0.106	0.321
HB (g/dl)	− 0.266*	**0.011**	− 0.074	0.490	0.154	0.147
PLT (x10^3^/ul)	− 0.413**	**0.000**	− 0.110	0.300	0.082	0.440
BM.LYM (%)	0.037	0.732	− 0.050	0.642	− 0.145	0.172

Spearman correlation coefficients; *: significant at *p* < 0.05; **: significant at *p* < 0.01; Significant values are in bold.

#### Studying the relationship between sADO levels and the other studied parameters among all mature B-cell lymphoma patients

Our data revealed a statistically significant positive relation between the increasing sADO level and risk stratification (*P* < 0.05) among all mature B-cell lymphoma patients. No significant relations were found between sADO levels and the other studied parameters.

### Studying the impact of elevated CD38 expression % and extracellular ADO production in the CLL patients’ group

#### Studying the effect of CD38 expression % in the CLL group by comparing CD38-negative Vs. CD38-positive patients regarding demographic data and characteristics

In our study, all sPD-L1, sPD-1, and sADO levels were significantly higher in the CD38-positive CLL patients when compared to the CD38-negative CLL patients (P-value < 0.01) (Table 5).

**Table 5:** Comparison between CD38-negative and CD38-positive CLL patients:

CLL patients’ group		CD38 Negative	CD38 Positive	P-value
		No. = 32	No. = 16	
Age (years)	Mean ± SD	56.37 ± 8.1	59.56 ± 8.37	0.210•
	Range	40–69	47–73	
Sex	Females	11 (34.4%)	3 (18.8%)	0.262*
	Males	21 (65.6%)	13 (81.3%)	
WBC (x 10^3^/ul)	Median (IQR)	60 (37.9–125)	82 (56–105)	0.319‡
	Range	10.5–531	16.2–428	
LYMPH (x 10^3^/ul)	Median (IQR)	49.5 (25–96.5)	69 (49–99.5)	0.225‡
	Range	6.6–480	11–358	
HB (g/dl)	Mean ± SD	9.87 ± 2.94	9.89 ± 3.08	0.986•
	Range	4.2–16.1	4.6–14	
PLT (x 10^3^/ul)	Median (IQR)	122.5 (83–164)	141.5 (68–243)	0.577‡
	Range	16–352	10–291	
BM.LYM (%)	Mean ± SD	81.47 ± 14.42	77.75 ± 11.32	0.372•
	Range	40–98	60–97	
P53 mutation	Negative	8 (72.7%)	5 (100.0%)	0.195*
	Positive	3 (27.3%)	0 (0.0%)	
sPD-L1 (ng/L)	Median (IQR)	106.7 (68.72–147.45)	422.5 (370.3–615.5)	0.000‡
	Range	27.97–1081	100.9–1600	
sPD-1 (ng/L)	Median (IQR)	99.34 (63–133.5)	461.5 (280–669.5)	0.000‡
	Range	19.38–280.5	148–1300	
sADO (ng/ml)	Median (IQR)	536 (296–621.5)	786.5 (678.5–1285.5)	0.000‡
	Range	73–777	569–1700	
Binet stages	A	13 (40.6%)	6 (37.5%)	0.900*
	B	8 (25.0%)	5 (31.3%)	
	C	11 (34.4%)	5 (31.3%)	
Risk stratification	Low	13 (40.6%)	6 (37.5%)	0.900*
	Intermediate	8 (25.0%)	5 (31.3%)	
	High	11 (34.4%)	5 (31.3%)	

*P* > 0.05: non-significant (NS); *P* < 0.05: significant (S); *P* < 0.01: highly significant (HS).

‡: Mann-Whitney test; •: Independent t-test; *: Chi-square test.

#### Studying the independent effect of lower vs. higher sADO level (regardless of CD38 expression %) on other studied parameters in the CLL group

When we used the mean of sADO level (694 ng/ml) in our study population as a cutoff to divide the CLL group into two sub-groups: one with sADO **<** 694 ng/ml and another with sADO ≥ 694 ng/ml, comparison between these two groups regarding demographic data and characteristics showed that the sub-group with sADO ≥ 694 ng/ml has a statistically significant increase in CD38 expression % (P-value < 0.01), sPD-1 (P-value < 0.01), sPD-L1 (*P* < 0.05). No difference regarding PLT count or HB was detected.

#### Correlation of sADO with sPDL1, sPD-1, and the other studied parameters among the CLL patients’ group

In the CLL patients’ group, our study showed a statistically positive correlation between sADO, sPD-1, and sPD-L1 serum levels (P-value < 0.01). No significant correlations were found between sADO and the other studied parameters among CLL patients.

### Studying the impact of elevated CD38 expression % and extracellular ADO production in the B-NHL patients’ group

#### Studying the effect of CD38 expression % in the B-NHL group by comparing CD38-negative Vs. CD38-positive patients regarding demographic data and characteristics

Our data revealed a statistically significant elevation in sPD-1 and sADO levels in CD38 positive B-NHL patients compared to CD38 negative B-NHL (P-value < 0.01). Furthermore, we found a statistically significant decrease in the mean HB level and median platelets count (P-value < 0.01) in CD38 positive B-NHL patients compared to CD38 negative B-NHL. Finally, there was a significant rise in the number of high-risk patients in CD38 positive B-NHL patients compared to CD38 negative B-NHL (P-value < 0.01). There was no statistically significant difference regarding the serum sPD-L1 level (Table 6).

**Table 6:** Comparison between CD38-negative and CD38-positive B-NHL patients:

B-NHL patients’ group		CD38 Negative	CD38 Positive	P-value
		No. = 10	No. = 32	
Age (years)	Mean ± SD	57.1 ± 11.52	52.28 ± 21.98	0.512•
	Range	40–68	23–80	
Sex	Females	1 (10.0%)	10 (31.3%)	0.182*
	Males	9 (90.0%)	22 (68.8%)	
WBC (x10^3^/ul)	Median (IQR)	51.75 (27.3–117)	30 (9.5–57.5)	0.193‡
	Range	9.3–134	3.01–175	
LYMPH (x10^3^/ul)	Median (IQR)	35 (19–103)	24 (10–44.5)	0.261‡
	Range	6–129	2–160	
HB (g/dl)	Mean ± SD	11.39 ± 2.62	8.94 ± 1.68	0.001•
	Range	5.2–13.7	6–14	
PLT (x10^3^/ul)	Median (IQR)	274 (233–307)	63 (50–112)	0.000‡
	Range	158–402	10–576	
BM.LYM (%)	Mean ± SD	59.1 ± 19.04	70.59 ± 23.56	0.169•
	Range	21–80	10–95	
sPD-L1 (ng/L)	Median (IQR)	183.25 (59–522.2)	168.92 (144.31–279.74)	0.595‡
	Range	21.51–1427	103.4–1124	
sPD-1 (ng/L)	Median (IQR)	67.1 (37–143)	178.63 (147–289)	0.003‡
	Range	21–680	110–1300	
sADO (ng/ml)	Median (IQR)	504.3 (343–567)	990.5 (742–1400.5)	0.000‡
	Range	77–614	553–1700	
Risk stratification	Low	0 (0.0%)	3 (9.4%)	0.001*
	Intermediate	10 (100.0%)	11 (34.4%)	
	High	0 (0.0%)	18 (56.3%)	

*P* > 0.05: Non-significant (NS); *P* < 0.05: Significant (S); *P* < 0.01: Highly significant (HS).

‡: Mann-Whitney test; •: Independent t-test; *: Chi-square test.

#### Studying the independent effect of lower vs. higher sADO level (regardless of CD38 expression %) on other studied parameters in the B-NHL group

When we used the mean of sADO level in our study population to divide the B-NHL patients into two sub-groups: one with sADO **<** 694 ng/ml and another with sADO ≥ 694 ng/ml, comparison between these two groups regarding demographic data and characteristics showed that the sub-group with sADO ≥ 694 ng/ml has a statistically significant increase in CD38 expression % (P-value < 0.01) and sPD-1 level (P-value < 0.01). Also, this group showed a statistically lower HB level (*P* < 0.05) and PLT count (P-value < 0.01). No significant difference was found regarding the serum sPD-L1 level.

#### Correlation between sADO with sPD-L1, sPD-1, and the other studied parameters among the B-NHL patients’ group

In the B-NHL patients’ group, sADO showed a statistically positive correlation with the sPD-1 level (P-value < 0.05) but no significant correlation with sPD-L1. Meanwhile, the sPD-L1 level showed a statistically positive correlation with the sPD-1 level (P-value < 0.05) but no significant correlation with sADO. However, the sPD-1 level showed a statistically positive correlation with sPD-L1 and sADO (P-value < 0.05). Additionally, a statistically significant negative correlation existed between sADO and Platelets count (P-value < 0.01) (Table 7).

**Table 7:** Correlation between sADO with sPD-L1, sPD-1, and the other studied parameters among the B-NHL patients’ group.

	sADO		sPD-L1		sPD-1	
	*r*	*p*	*r*	*p*	*r*	*p*
sPD-L1 (ng/L)	0.116	0.465			0.312*	**0.044**
sPD-1 (ng/L)	0.324*	**0.037**	0.312*	**0.044**		
sADO (ng/ml)			0.116	0.465	0.324*	**0.037**
Age (years)	− 0.128	0.418	0.079	0.619	− 0.088	0.581
WBC (x10^3^/ul)	− 0.212	0.178	− 0.213	0.175	− 0.204	0.195
LYMPH (x10^3^/ul)	− 0.240	0.126	− 0.253	0.106	− 0.259	0.098
HB (g/dl)	− 0.223	0.155	0.165	0.297	− 0.102	0.519
PLT (x10^3^/ul)	− 0.490**	**0.001**	0.058	0.714	− 0.171	0.280
BM.LYM (%)	0.301	0.053	− 0.082	0.606	− 0.039	0.808

Spearman correlation coefficients; *: significant at *p* < 0.05; **: significant at *p* < 0.01; Significant values are in bold.

## Discussion

In the closed hypoxic BM niche, cross-talk among the distinct cellular components and tumor cells within the tumor microenvironment gives preference to molecular pathways that produce extracellular ADO, which enhances tumor aggressiveness and leads to tumor cell survival. This occurs by binding extracellular ADO to purinergic receptors, which leads to the active complex formation that functions as autocrine/paracrine signals with immune regulatory activities^16^.

Adenosine (ADO) is produced from the breakdown of nucleotides like ATP, ADP, ADPR, AMP, and NAD+. This occurs through two pathways: the Conical Pathway, where CD39 converts ATP to ADP and then AMP via CD73, and the Non-Conical Pathway, where CD203a/PC-1 converts ADPR (generated from NAD^+^ by CD38) or ATP directly to AMP via CD73. While ADO levels are typically low under normal conditions, they can rise significantly during tissue injury, ischemia, hypoxia, inflammation, trauma, and cancer. This increase activates specific adenosine receptors (ADOR) on immune cells, resulting in immunosuppressive effects^17,18^.

Recent research postulated that enhancement of extracellular ADO in tumors expressing CD38 mediates not only immunosuppressive effects but also a tumor escape mechanism by increasing PD-1 expression on CD8 + cytotoxic T cells. This, via binding with its ligand (PD-L1), suppresses the CD8 + cytotoxic T cells’ activity and immunosurveillance^19^. Considering this role of extracellular ADO in regulating inflammation and immunogenicity, evidence emphasizes the Non-Conical Pathway (CD38/CD203a/CD73 ectoenzymatic pathway independent of CD39) could constitute a novel strategy for tumor evasion, implying that these enzymes may represent ideal targets for antibody-mediated therapy^16–20^.

Altogether, this evidence suggests a critical role of extracellular ADO in tumorigenesis and the PD-1/PD-L1 axis dysregulation in CD38-positive cancers; however, this approach has yet to be explored in depth in mature B-cell neoplasm patients.

Armed with these compelling data, we meticulously crafted our study to investigate the potential influence of heightened CD38 expression and extracellular ADO production on various demographic, clinical, and hematological parameters in mature B-cell lymphoma patients, including CLL and B-NHL. Our focus was on their association with the soluble counterparts of the PD-1/PD-L1 axis.

Our comprehensive study, which thoroughly investigated the link between elevated CD38 expression and extracellular ADO production with the PD-1/PD-L1 axis, uncovered a positive correlation between sPD-L1, sPD-1, sADO, and CD38 expression in all mature B-cell lymphoma patients. We also noted a significant increase in sPD-L1, sPD-1, and sADO levels in CD38-positive mature B-cell lymphoma patients. Moreover, sADO level (regardless of CD38 expression) was positively associated with sPD-1, and sPD-L1 in all studied patients’ groups. These findings comprehensively support the hypothesized link between elevated CD38 expression, extracellular ADO production, and the PD-1/PD-L1 axis in all mature B-cell lymphoma patients.

The immune regulatory molecules PD-1 and PD-L1 have two forms of expression: membrane-bound and soluble. The soluble form of these molecules (sPD-1 and sPD-L1) results from the proteolytic cleavage of the membrane-bound forms^21^. Five splice variants of PD-1 mRNA transcripts have been cloned from human peripheral blood mononuclear cells. The PD-1 Deltaex3 variant encodes the sPD-1, which is usually undetectable in healthy individuals. sPD-L1 is detectable in supernatants from mPD-L1 positive cell lines rather than those from mPD-L1 negative cell lines^22^. Many regulatory immune cells can generate sPD-L1, emphasizing the regulatory mechanism of this molecule in the tumor microenvironment. A “state of balanced immune homeostasis” exists between sPD-1 and sPD-L1 molecules, affecting T cell activation/inhibition and impacting cancer immune evasion and outcomes^23^.

Recent studies have proved that soluble PD-1 (sPD-1) and soluble PD-L1 (sPD-L1) exhibit similar activity to their membrane-bound forms, transmitting signals between immune cells and influencing their functions. Evidence indicates that sPD-1 and sPD-L1 are readily detectable in clinical settings, reflecting both that are released from tumor cells and immune regulatory cells and may significantly impact tumor development and immune responses. As such, they hold potential as biomarkers and insights for treatment strategies, including immunotherapy for malignant tumors^23,24^.

In coordinance with our findings, Chen et al. 2018 reported an up-regulation of CD38 in lung cancer and melanoma cells from murine models chronically exposed to PD-1/PD-L1inhibitors, which enhances ADO signaling, suppressing the CD8^+^ T-cell function, thus allowing an escape pathway from the immune system and used targeted therapy^25^. Also, Costa et al. 2021 reported in preclinical models of different solid tumors, such as HCC and MM patients, that the resistance to the anti-PD-1/PD-L1 antibody is convoy by CD38 up-regulation, which mediates its immune-suppression effect via enhancing extracellular ADO^11^. Additionally, Khan et al. 2020 stated the positive correlation between the circulating levels of sPD-1 and sPD-L1 in multiple solid tumors, for instance, DLBCL, CLL, NSCLC, HCC, and metastatic melanoma^23^.

To compare the impact of the elevated CD38 expression and extracellular ADO production on the PD-1/PD-L1 axis between the CLL and B-NHL group, our study revealed a higher sPD-L1 level in the CLL vs. B-NHL group. In the CLL group, there was also a higher PD-L1 level in CD38-positive patients and patients with sADO ≥ 694 ng/ml. Nevertheless, this difference was not observed in B-NHL patients. Based on the findings, we believe that the elevated serum levels of sPD-L1 in the CLL group are mostly related to the disease pathophysiology as a slowly increasing and resistant to apoptosis lymphoid cells able to escape immunosurveillance^26^. So, this group’s higher WBC and lymphocyte counts could also explain this finding.

To further explain this point, a summary review of different immunohistochemical studies demonstrated that although DLBCL has a high expression level of PD-1 and PD-L1, other B-cell lymphomas such as LPL, MCL, and MZL tumor cells are usually negative for PD-L1. On the other hand, they reported an increased PD-1/PD-L1 expression in CLL cases as a known mechanism of immune escape in this category^27–29^. Additionally, Yang and Hu 2019 reported the variability in PD-L1 expression in lymphoma cells, the highest found in DLBCL, followed by small lymphocyte lymphoma/CLL, and lowest in inactive follicular lymphoma, suggesting that PD-L1 may be associated with lymphoma invasiveness^30^.

To study the effect of CD38 expression on other laboratory parameters, we found that all CD38-positive mature B-cell lymphoma patients had significant thrombocytopenia compared to CD38-negative patients. We also detected substantial thrombocytopenia and anemia in CD38-positive B-NHL patients compared to CD38-negative B-NHL patients. However, we did not observe these findings in the CLL group.

To compare these previous findings with the independent effect of Extracellular ADO in our study, we found that sADO was markedly elevated in all mature B-cell lymphoma patients since the normal range of plasma ADO levels was reported in previous literature to range from 0.04 to 0.2 micromoles micromoles/L, equivalent to 0.149–0.748 ng/ml^31^. At higher sADO levels (sADO ≥ 694 ng/ml), there were higher degrees of thrombocytopenia and anemia in all mature B-cell lymphoma patients and the B-NHL group in comparison to lower sADO levels (sADO < 694 ng/ml). Besides, sADO level was inversely correlated with the HB level and PLT count in all mature B-cell lymphoma patients and with the PLT count only in the B-NHL group. However, we did not observe these findings in the CLL group.

These findings suggest that the elevated CD38 expression and extracellular ADO production play a role in anemia and thrombocytopenia associated with the mature B-cell neoplasm, which was more pronounced in the B-NHL group.

To further explain the CD38-mediated ADO production, Chen et al. 2018, stated that CD38 upregulation on tumor cells is induced by inflammatory cytokine production, revealing a positive correlation between immunohistochemical (IHC) staining for CD38 on lung cancer specimens and the inflamed microenvironment. Additionally, they stated that CD38 mediates its suppressive effect via extracellular ADO enhancement acting through ADO receptors^25^. Assmann et al. 2020 demonstrated that the environment surrounding the tumor niche is characterized by unusually high concentrations of purinergic molecules, such as ATP, ADP, and ADO, that favor tumor progression^32^. Other studies have shown that the concentration of extracellular ADO is markedly increased during stressful situations like hypoxia, ischemia, and cellular damage in the tumor microenvironment, which, in turn, stimulates the growth of many cancers by activating the A2B-ADO receptors on different cellular component^33,34^. By acting on erythrocytes through the A2B receptor, ADO increases erythrocyte 2,3-BPG levels and enhances O2-releasing capacity and VEGF production by endothelial cells in the tumor microenvironment, which helps promote tumor angiogenesis^35^.

Also, a recent study by Mikadar et al., 2024, proved that extracellular ADO at high concentrations mediates a decrease in erythroid proliferation and attenuates erythroid maturation by activating A3 and A2B receptors. ADO signaling through these receptors inhibits the proliferation of erythroid precursors by activating the P53 pathway and hampers terminal erythroid maturation^13^. Further, many recent studies have found that ADO binding of A2 subtypes (A2A or A2B) in platelets leads to the consequent elevation of intracellular cyclic AMP, an inhibitor of platelet activation. This, in turn, inhibits platelet activation in response to ADP; this effect is accentuated further during ischemic and inflammatory conditions^36,37^.

Finally, to study the effect of the elevated CD38 expression and extracellular ADO production on risk stratification and other prognostic markers, our study convoy risk stratification according to the Binet staging system for the CLL group and the International Prognostic Index and ECOG performance scale for the B-NHL group. CD38 positivity with higher sADO levels was more evident in patients with higher risk stratification in all mature B-cell lymphoma and the B-NHL group. This finding was not detected when studying the CLL group. Also, at higher sADO levels (sADO ≥ 694 ng/ml), there was an increased number of patients with P53 deletion, which is considered a poor prognostic marker in mature B-cell lymphoma^38^. In our study, sPD-1 and sPD-L1 levels did not correlate with risk stratification in any studied groups. These findings suggest that the elevated CD38 expression and extracellular ADO production contribute to the neoplastic process and may have a poor predictive value in mature B-cell neoplasm.

In agreement with our findings, many recent studies, such as Haq et al. 2020, and Wada et al. 2021, have shown that high CD38 expression is an independent adverse prognostic factor associated with poor clinical outcomes when compared to low CD38 expression in mature B cell neoplasms such as CLL and DLBCL cells and can be used to predict outcomes and design risk-adapted therapies^39,40^.

Additionally, Nakamura et al., 2020, demonstrated that ADO could be a potential target for leveraging anti-lymphoma immunity. ADO acts as a “do not eat me signal,” allowing tumors to escape the immune system. They also found that excessive expression of CD39, an enzyme involved in ADO production, negatively impacts the prognosis of patients with DLBCL and the effectiveness of rituximab in preclinical studies^41^. Furthermore, other research demonstrated that ADO has a negative prognostic impact on the growth of different solid tumors, such as colorectal cancer^42^ and melanoma^43^. Recent data additionally supported that targeting the ADO production pathway is a successful antitumor immunotherapy, especially targeting A2AR, CD39, and CD73^34,44–46^.

## Conclusion

This study examined the potential effects of CD38 expression and increased extracellular ADO production in B-cell lymphoma on various hematological and clinical parameters and their possible connection to the PD-1/PD-L1 axis. Our findings highlight the role of extracellular ADO in the neoplastic process of B-cell neoplasms, particularly in patients with B-NHL. Furthermore, we suggest that targeting the pathway of CD38-induced ADO formation may serve as a promising therapeutic approach, with diverse effects on mature B-cell neoplasms, including interactions with the PD-1/PD-L1 axis and the management of associated conditions such as thrombocytopenia and anemia.

Further studies are necessary to clarify the pathophysiology of CD38-induced ADO formation and strengthen the link between CD38 expression and the output of ADO and PD-1/PD-L1 in B-cell neoplasms. These studies will be crucial for developing effective therapeutic strategies and improving patients’ selection for ADO pathway modulators.

## Data Availability

Sequence data that support the findings of this study have been deposited in an online repository available for any one with the following URL: https://drive.google.com/file/d/1VvDYtYVy_1xcRDATJxVmpX9Wqb-V5lyn/view?usp=sharing.

## Abbreviations

CLL: Chronic lymphocytic leukemia
B-NHL: Non-Hodgkin lymphoma
sADO: Serum adenosine
sPD-L1: Serum programmed death ligand-1
sPD-1: Serum programmed death-1
BM.LYM: Bone marrow lymphocytes
IPT: Immunophenotyping
HCC: Hepatocellular carcinoma
MM: Multiple myeloma
DLBCL: Diffuse large B-cell lymphoma
NPC: Nasopharyngeal carcinoma
NSCLC: Non-small cell lung carcinoma
ECOG: Eastern cooperative oncology group performance status
ATP: Adenosine triphosphate
ADP: Adenosine diphosphate
AMP: Adenosine monophosphate
ADPR: Adenosine diphosphate ribose
ADOR: Adenosine receptors
